# *KAE1* Allelic Variants Affect TORC1 Activation and Fermentation Kinetics in *Saccharomyces cerevisiae*

**DOI:** 10.3389/fmicb.2019.01686

**Published:** 2019-07-31

**Authors:** Eduardo I. Kessi-Pérez, Francisco Salinas, Asier González, Ying Su, José M. Guillamón, Michael N. Hall, Luis F. Larrondo, Claudio Martínez

**Affiliations:** ^1^Departamento de Ciencia y Tecnología de los Alimentos, Universidad de Santiago de Chile (USACH), Santiago, Chile; ^2^Centro de Estudios en Ciencia y Tecnología de Alimentos (CECTA), Universidad de Santiago de Chile (USACH), Santiago, Chile; ^3^Millennium Institute for Integrative Biology (iBio), Santiago, Chile; ^4^Instituto de Bioquímica y Microbiología, Facultad de Ciencias, Universidad Austral de Chile (UACH), Valdivia, Chile; ^5^Biozentrum, University of Basel, Basel, Switzerland; ^6^Departamento de Biotecnología de los Alimentos, Instituto de Agroquímica y Tecnología de Alimentos (IATA), Consejo Superior de Investigaciones Científicas (CSIC), Valencia, Spain; ^7^Departamento de Genética Molecular y Microbiología, Facultad de Ciencias Biológicas, Pontificia Universidad Católica de Chile, Santiago, Chile

**Keywords:** *Saccharomyces cerevisiae*, TORC1 pathway, natural variation, microculture, fermentation, tRNA modification

## Abstract

The eukaryotic domain-conserved TORC1 signalling pathway connects growth with nutrient sufficiency, promoting anabolic processes such as ribosomal biogenesis and protein synthesis. In *Saccharomyces cerevisiae*, TORC1 is activated mainly by the nitrogen sources. Recently, this pathway has gotten renewed attention but now in the context of the alcoholic fermentation, due to its key role in nitrogen metabolism regulation. Although the distal and proximal effectors downstream TORC1 are well characterised in yeast, the mechanism by which TORC1 is activated by nitrogen sources is not fully understood. In this work, we took advantage of a previously developed microculture-based methodology, which indirectly evaluates TORC1 activation in a nitrogen upshift experiment, to identify genetic variants affecting the activation of this pathway. We used this method to phenotype a recombinant population derived from two strains (SA and WE) with different geographic origins, which show opposite phenotypes for TORC1 activation by glutamine. Using this phenotypic information, we performed a QTL mapping that allowed us to identify several QTLs for TORC1 activation. Using a reciprocal hemizygous analysis, we validated *GUS1*, *KAE1*, *PIB2*, and *UTH1* as genes responsible for the natural variation in the TORC1 activation. We observed that reciprocal hemizygous strains for *KAE1* (ATPase required for t^6^A tRNA modification) gene showed the greatest phenotypic differences for TORC1 activation, with the hemizygous strain carrying the SA allele (*KAE1*^*SA*^) showing the higher TORC1 activation. In addition, we evaluated the fermentative capacities of the hemizygous strains under low nitrogen conditions, observing an antagonistic effect for *KAE1*^*SA*^ allele, where the hemizygous strain containing this allele presented the lower fermentation rate. Altogether, these results highlight the importance of the tRNA processing in TORC1 activation and connects this pathway with the yeasts fermentation kinetics under nitrogen-limited conditions.

## Introduction

The TORC1 signalling pathway is conserved in eukaryotes and connects growth with nutrient sufficiency, promoting anabolic processes such as ribosomal biogenesis and protein synthesis. TOR was originally discovered in *Saccharomyces cerevisiae* (Heitman et al., 1991), and since then its study has been extended to other model organisms, including *Arabidopsis thaliana* (AtTOR), *Caenorhabditis elegans* (CeTOR), *Drosophila melanogaster* (dTOR), and zebrafish (zTOR), as well as mammals (mTOR) (Crespo and Hall, 2002).

In *S. cerevisiae*, two kinases (Tor1 and Tor2) form part of two protein complexes (Tor1 or Tor2 in TORC1, but only Tor2 in TORC2), where only TORC1 is inhibited by rapamycin (Loewith et al., 2002; Loewith and Hall, 2011). In this yeast, TORC1 is activated mainly by the availability of nitrogen sources in the culture medium, increasing its activity as result of the medium supplementation with ammonium or amino acids, or even after a treatment with cycloheximide (causes an increase in the intracellular amino acids levels due to the translation decay). On the contrary, TORC1 activity decreases after nitrogen depletion in the environment (Broach, 2012).

Recently, the TORC1 signalling pathway activation has gotten renewed attention but now in the context of the alcoholic fermentation, due to its key role in the regulation of nitrogen metabolism (Tesniere et al., 2015). *S. cerevisiae* is the main species responsible for the transformation of grape must into wine by the process known as alcoholic fermentation (Pretorius, 2000). Deficiency of nitrogen sources in the grape must is a main problem for the wine industry, because the reduced fermentation rate leads to sluggish or stuck fermentations, generating economic losses in the industry (Taillandier et al., 2007). This occurs mainly because nitrogen sources are one of the most important factors regulating biomass during fermentation, which impacts fermentation rate (Varela et al., 2004).

There are two main proximal effectors of TORC1: the Tap42-PP2A phosphatase complex and the Sch9 kinase (Loewith and Hall, 2011; Hughes Hallett et al., 2014). When nitrogen sources are available in the environment TORC1 is active and phosphorylates its proximal effectors, activating the branch associated with the Sch9 protein, promoting anabolic processes and subsequent cell growth. Protein synthesis and ribosome biogenesis are the key processes involved in cell growth, which are partly mediated by the activation of genes encoding ribosomal proteins (Oliveira et al., 2015). In addition, TORC1 phosphorylates other proximal effectors such as Ypk3, an AGC kinase that in turn phosphorylates the Rps6 ribosomal protein (Gonzalez et al., 2015; Yerlikaya et al., 2016).

Although the distal and proximal effectors downstream TORC1 are well known, it is not fully understood the mechanism by which TORC1 is activated by nitrogen sources (Conrad et al., 2014; Gonzalez and Hall, 2017). How nitrogen availability information is transmitted to the TORC1 complex is unclear (Kim et al., 2012; Ljungdahl and Daignan-Fornier, 2012; Gonzalez and Hall, 2017). It has been hypothesised that amino acids are detected intracellularly, either at the cytoplasm or during its passage through the vacuolar membrane, although it has not been ruled out the possibility of some type of detection at the cytoplasmic membrane. In addition, there is debate on whether all amino acids are sensed independently or if only a few key amino acids are sensed (Conrad et al., 2014).

The EGO complex (EGOC) has been described as part of a mechanism of TORC1 activation that is amino acid-dependent. The main components of the EGOC are Gtr1 an Gtr2, both GTPases (Hatakeyama and De Virgilio, 2016; Powis and De Virgilio, 2016). However, how EGOC senses the amino acids is unknown, being the exception leucine, which is sensed through the leucyl-tRNA synthetase (LeuRS) (Bonfils et al., 2012). EGOC-dependent activation of TORC1 occurs rapidly but transiently by both preferred and non-preferred nitrogen sources. However, only preferred sources promote an EGOC-independent and sustained activation, coupled with intracellular glutamine accumulation (Stracka et al., 2014). In addition, the signalling defect of TORC1 in ammonium deprivation is not supressed by a constitutive activity of EGOC (Binda et al., 2009).

Therefore, the current evidence points out the existence of an EGOC-independent TORC1 activation mechanism, in which have not been determined all the participating proteins (Chantranupong et al., 2015; Gonzalez and Hall, 2017). In this regard, there are only a few suggested agents such as Pib2, a vacuolar membrane protein of unknown function (Kim and Cunningham, 2015; Michel et al., 2017; Tanigawa and Maeda, 2017; Varlakhanova et al., 2017; Ukai et al., 2018; Sullivan et al., 2019). It has also been suggested that free tRNAs regulate the activity of TORC1, although with a different mechanism than the one proposed for leucine (Kamada, 2017).

In a previous work, we developed a methodology for the indirect evaluation of EGOC-independent TORC1 activation by a nitrogen upshift experiment, using the luciferase gene (as a reporter) under the control of the *RPL26A* gene promoter region (Kessi-Perez et al., 2019). Using this method, we showed the existing of natural variation in the activation of TORC1 signalling pathway in yeast, opening the possibility of using this methodology to investigate the molecular and genetic basis of TORC1 activation utilising high throughput approaches like comparative genomics, BSA or linkage analysis, which requires evaluating the phenotype of many strains.

In this work, we performed a QTL mapping analysis using the phenotypic information obtained from a recombinant population derived from two strains (SA and WE) that showed differences in TORC1 activation by glutamine, using the abovementioned methodology for phenotyping. We identified several QTLs and validated *GUS1*, *KAE1*, *PIB2*, and *UTH1* as genes responsible for the natural variation in the TORC1 activation. The greatest phenotypic differences were observed between the reciprocal hemizygous strains for *KAE1* (ATPase required for t^6^A tRNA modification), reinforcing the importance tRNAs in TORC1 activity. The allelic differences in this gene also impact on the fermentative capacities of the analysed yeast strains, resulting in a direct connection between TORC1 activation and yeasts fermentation kinetics under nitrogen-limited conditions.

## Materials and Methods

### Yeast Strains

#### Parental, Hybrid and Segregant Strains

Parental strains Y12 (“SA”) and DBVPG6765 (“WE”) carrying the *Luc-URA3* construct instead the endogenous *RPL26A* ORF, as well as the hybrid between them (“SA×WE”), were previously generated (Kessi-Perez et al., 2019). A recombinant population utilised in this work is composed by 96 segregants derived from the SA×WE cross, and it was previously described and genotyped (Cubillos et al., 2011). In each segregant of this population, we replaced the endogenous *RPL26A* ORF by the *Luc-URA3* construct, as previously described (Kessi-Perez et al., 2019). All the strains are listed in Supplementary Table 1.

#### Reciprocal Hemizygous Strains

The reciprocal hemizygotes were generated as previously described (Salinas et al., 2012; Jara et al., 2014; Kessi-Perez et al., 2016). Initially, knockouts of 17 candidate genes were generated from each parental strain (SA and WE), which already were carrying the *Luc-URA3* construct. For both strains, the *HygMX* (hygromycin resistance) gene was used as a selection marker. The transformations were carried out as previously described (Jara et al., 2014). The mutated parental strains were crossed to generate diploid reciprocal hemizygous strains and confirmed using *MAT* locus PCR (Huxley et al., 1990). All these strains are listed in Supplementary Table 1.

### Evaluation of the TORC1 Pathway Activation

#### Microculture

TORC1 activation was evaluated in the strains carrying the *Luc-URA3* reporter [controlled by the *RPL26A* promoter (*P*_*RPL26A*_)] by monitoring simultaneously the luminescence (Lum) and optical density at 600 nm (OD_600_) of the cells in microculture conditions, as previously described (Salinas et al., 2016; Kessi-Perez et al., 2019). Briefly, we performed a nitrogen upshift experiment, where the strains were grown at 30°C in 96-well plates containing 300 μL of yeast minimal medium (20 g/L glucose and 1.7 g/L yeast nitrogen base without amino acids and without ammonium sulphate) with proline (0.5 mg/mL) as the only nitrogen source (YMM+Pro), supplemented with luciferin (1 mM), until OD_600_ ∼0.8. Then, 10 μL of glutamine (15 mg/mL; 0.5 mg/mL final concentration) were added (Kessi-Perez et al., 2019). Luminescence was measured using 10 min intervals in a Synergy HTX microplate reader (Biotek, United States) up to 12 h. All microculture experiments were carried out in three independent biological replicas.

#### Immunoblot

The direct evaluation of the TORC1 pathway activation was carried out assessing the phosphorylation in a nitrogen upshift experiment of the ribosomal protein Rps6 (Gonzalez et al., 2015). Briefly, strains were grown until OD_600_ ∼0.8 in flasks containing 50 mL of YMM+Pro medium and then 700 μL of glutamine (25 mg/ml; 0.5 mg/mL final concentration) were added. Samples were taken to perform protein extraction and subsequent immunoblot at different time points (0, 5, 15, and 30 min), as previously described (Gonzalez et al., 2015). Antibodies used were the phospho-Ser235/Ser236-S6 (Cell Signaling Technology Cat# 2211, RRID:AB_331679), RPS6 (Abcam Cat# ab40820, RRID:AB_945319) and peroxidase-Monoclonal Mouse Anti-Rabbit IgG (Jackson ImmunoResearch Labs Cat# 211-032-171, RRID:AB_2339149).

### Evaluation of Fermentative Performance

#### Synthetic Must Composition

The fermentation experiments were conducted in synthetic must (SM), which mimics the natural grape must but with a defined composition. The SM was prepared as previously described (Riou et al., 1997), with some modifications. The SM contains: 200 g/L of sugar (100 g/L glucose and 100 g/L fructose), malic acid 5 g/L, citric acid 0.5 g/L, tartaric acid 3 g/L, KH_2_PO_4_ 0.75 g/L, K_2_SO_4_ 0.5 g/L, MgSO_4_ 0.25 g/L, CaCl_2_ 0.16 g/L, NaCl 0.2 g/L, trace elements [MnSO_4_ 4 mg/L, ZnSO_4_ 4 mg/L, CuSO_4_ 1 mg/L, KI 1 mg/L, CoCl_2_ 0.4 mg/L, H_3_BO_3_ 1 mg/L, and (NH_4_)_6_Mo_7_O_24_ 1 mg/L] and vitamins (myo-inositol 20 mg/L, calcium pantothenate 1.5 mg/L, nicotinic acid 2 mg/L, thiamine hydrochloride 0.25 mg/L, pyridoxine hydrochloride 0.25 mg/L, and biotine 0.003 mg/L). The composition of nitrogen source in the SM is 40% ammonium and 60% amino acids, and its concentration was modified at different levels: 60, 140, and 300 mg/L of yeast assimilable nitrogen (YAN) (named SM60, SM140 and SM300, respectively). The final pH of the SM was adjusted to 3.3 with sodium hydroxide and then the SM was sterilised by filtration through a 0.22 μm pore size membrane filter. The composition of amino acids in 1 L of stock was: l-tyrosine 1.5 g, l-tryptophan 13.4 g, l-isoleucine 2.5 g, aspartic acid 3.4 g, glutamic acid 9.2 g, l-arginine 28.3 g, l-leucine 3.7 g, l-threonine 5.8 g, glycine 1.4 g, l-glutamine 38.4 g, l-alanine 11.2 g, l-valine 3.4 g, l-methionine 2.4 g, l-phenylalanine 2.9 g, l-serine 6 g, l-histidine 2.6 g, l-lysine 1.3 g, l-cysteine 1.5 g and l-proline 46.1 g, which correspond to 13.75 g/L of assimilable nitrogen.

#### Microculture Fermentations

The growth parameters of the strains under fermentative conditions were evaluated by monitoring the OD_600_ of the cells in microculture conditions. Strains were inoculated at 10^6^ yeast cells/mL and grown at 28°C in 96-well plates containing 250 μL of SM60, SM140 or SM300. The OD_600_ was measured using 30 min intervals in a SPECTROstar Nano^®^ microplate reader (BGM Labtech, Offenburg, Germany). All microculture experiments were carried out in three independent biological replicas.

#### Microscale Fermentations

The fermentation kinetics of the strains were evaluated by monitoring CO_2_ production using a microscale fermentation method. The strains were inoculated at 10^6^ yeast cells/mL and grown at 28°C in 15 mL tubes containing 10 mL of SM60, SM140 or SM300, and permitting the CO_2_ release. Fermentations were followed by CO_2_ production, which was monitored by the weight loss of the fermentation tubes. Three tubes filled with 10 mL SM without inoculation were used as controls for the evaporation weight loss (EWL). The weight loss (WL) was calculated as:

$$\mathrm{𝑊𝐿}=W_{0}-W_{t}-\mathrm{𝐸𝑊𝐿}$$

The fermentations were considered as finished when WL stops to increase. The loss of CO_2_ was calculated from the weight loss of tubes as previously described (Kessi-Perez et al., 2016). All microscale experiments were carried out in three independent biological replicas.

### Statistical Analysis of Curves

#### Luminescence Curves

The luminescence curves parameters (maximum luminescence time, maximum luminescence value and area under the luminescence curve) were extracted from high-density luminescence curves with the “Area under curve” option of the “Analyze Data” menu in Graph Pad Prism 7.04 software as previously described (Kessi-Perez et al., 2019), and compared between strains using Welch two sample *t*-tests, performed using R software (R Core Team, 2013).

#### Growth Curves

Growth parameters (relative fitness variables) for each strain were calculated as previously described (Warringer et al., 2011; Kessi-Perez et al., 2016, 2019). Briefly, lag time of proliferation, rate of proliferation (population doubling time) and efficiency of proliferation (population density change) were extracted from high-density growth curves using the “Gompertz growth equation” (Yin et al., 2003), utilising the “Analyze Data” option from the menu in Graph Pad Prism 7.04 software. Statistical analysis of these parameters were performed using R software and consisted in Welch two sample *t*-tests (R Core Team, 2013).

### QTL Mapping

QTL mapping was performed as previously described (Cubillos et al., 2011; Jara et al., 2014; Kessi-Perez et al., 2016), using the R/qtl package in R software (Broman et al., 2003). Briefly, the LOD scores were estimated using a non-parametric model, in which the significance of each region was determined from permutations, using the “scanone” function of R/qtl. The phenotypic values were permuted 1000 times, recording the maximum LOD score each time. Statistically significant QTLs were those with a *p*-value < 0.05 (which means that their LOD score was greater than the 0.05 of the 1000 LOD scores permuted) and marginally significant QTLs those with a *p*-value < 0.15. For each QTL obtained, a better position was estimated using the “refineqtl” function of R/qtl. Then, the percentage of phenotypic variance explained by each QTL was determined using the “addint” function of R/qtl, which utilises the following formula (where “n” represents the sample size):

$$\mathrm{𝐸𝑥𝑝𝑙𝑎𝑖𝑛𝑒𝑑}\,\mathrm{𝑝ℎ𝑒𝑛𝑜𝑡𝑦𝑝𝑖𝑐}\,\mathrm{𝑣𝑎𝑟𝑖𝑎𝑛𝑐𝑒}=100\times \left(1-10^{\left(-\frac{2\,\mathrm{𝐿𝑂𝐷}}{n}\right)}\right)$$

### Bioinformatic Tools

The nucleotide sequences of the loci under study for each parental strain were obtained from the *Saccharomyces* Genome Resequencing Project (SGRP) database, available online^1^ (Bergstrom et al., 2014), from where we downloaded the sequence information as FASTA files. The nucleotide sequences were translated into amino acid sequences using the online bioinformatic tool ExPASy^2^, belonging to the Swiss Institute of Bioinformatics (SIB), utilising the standard genetic code and selecting the open reading frame that comprises the entire sequence from the first ATG codon. Protein sequences alignments between parental strains were performed using the online bioinformatic tool ClustalOmega^3^, belonging to The European Bioinformatics Institute (EBI), and using the default parameters provided by the software. The prediction of the effect over the protein for each genetic variant existing on the parental strains was carried out using the online tool PROVEAN^4^, belonging to the J. Craig Venter Institute (JCVI). We used the “PROVEAN Protein” option, the “NCBI nr, September 2012” database and the default parameters provided by the software; the statistical cut-off value used to consider a predicted variant as deleterious corresponded to a PROVEAN score < 2.500.

## Results

### Mapping Genetic Variants Affecting TORC1 Activation

In a previous work, we developed a microculture-based methodology for indirect evaluation of TORC1 activation in a nitrogen upshift experiment (Kessi-Perez et al., 2019). Based on this method, we observed phenotypic diversity in the TORC1 signalling pathway activation between representative strains of the clean lineages described so far in *S. cerevisiae* (Liti et al., 2009), with the major differences between the Y12 (Sake, SA) and DBVPG6765 (Wine European, WE) strains. Initially, we confirmed these results using a different microplate reader, observing the same luminescence pattern for these two strains (Supplementary Figure 1). Subsequently, we took a recombinant population composed of 96 haploid segregants sporulated from the diploid hybrid strain coming from the SA×WE cross (Cubillos et al., 2011) and we transformed each segregant with the *Luc-URA* construct to replace the *RPL26A* gene. Thus, the luciferase gene is now under the control of the endogenous *RPL26A* promoter, a downstream target of the TORC1 pathway. This new population was phenotyped in triplicate through nitrogen-upshift experiments in microculture conditions, in which cells are first grown in proline as unique nitrogen source and then a pulse of glutamine is given. During the nitrogen-upshift experiments, we quantified three parameters associated with the luminescence curves (maximum luminescence time, maximum luminescence value and area under the luminescence curve) at three different time intervals (0 to 12 h, 0 to 4 h, and 4 to 12 h).

**FIGURE 1:**
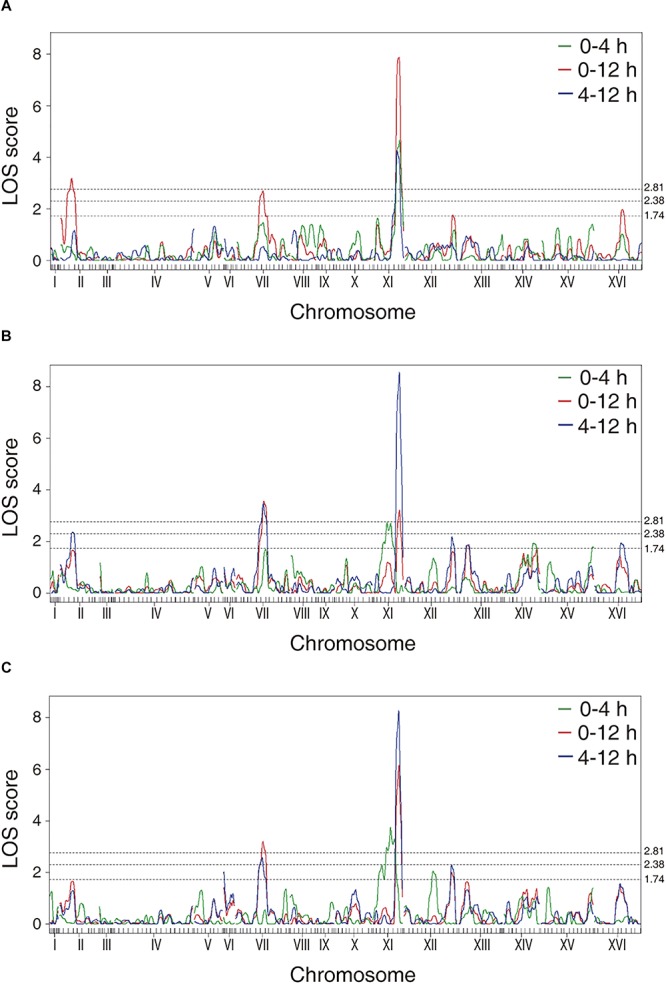
LOD scores for the mapped QTLs. The graphs represent the results obtained for the phenotypes of **(A)** time in which the maximum luminescence is obtained, **(B)** maximum luminescence and **(C)** area under the curve of luminescence. The dotted lines correspond to the LOD scores of 2.81 (*p*-value < 0.05), 2.38 (*p*-value < 0.15), and 1.74 (*p*-value < 0.50).

The phenotypic values obtained (Supplementary Figure 2 and Supplementary Table 2) were used to perform a QTL mapping by linkage analysis, following a protocol previously used by Kessi-Perez et al. (2016), in which the phenotype of each segregant is correlated with its genotype to find genomic regions (QTLs) involved in the trait under study. Five QTLs were obtained, in chromosomes II (Q1), VII (Q2 and Q3) and XI (Q4 and Q5) (Figure 1 and Table 1). A window of 15 kb was taken upstream and downstream for each QTL peak, among which 17 candidate genes were selected (Table 2). This selection was based on the existence of differences in the amino acid sequences between parental strains and matching at least one of the following criteria: (i) deletion or overexpression of the gene causes sensitivity or resistance to rapamycin, a phenotype commonly used for TORC1 evaluation (Loewith and Hall, 2011); (ii) the protein is involved in tRNA processing, because it has been suggested that tRNAs may regulate TORC1 (Kamada, 2017); and (iii) the protein is found in the plasma membrane or in the vacuole, because these are possible places where nitrogen source could be sensed (Ljungdahl and Daignan-Fornier, 2012; Conrad et al., 2014; Rodkaer and Faergeman, 2014). The mentioned analysis was done using the information of the SGD (*Saccharomyces* Genome Database^5^) as a reference. Altogether, the TORC1 activation showed a great phenotypic diversity in the recombinant population, which allowed us the QTL mapping for this phenotype and the selection of candidate genes related to the activation of the pathway.

**TABLE 1:** Linkage analysis for TORC1 activation.

**Phenotype**	**QTL**	**Position**	**LOD score**	***p*-value**	**Explained phenotypic variance**
Time_0–4_	Q5	XI.505	4.66	0.003^*^	21.5%
Time_0–12_	Q1	II.309	3.19	0.024^*^	8.3%
	Q2	VII.612	2.70	0.069^ms^	1.1%
	Q5	XI.505	7.88	0.000^*^	33.3%
Time_4–12_	Q5	XI.505	4.27	0.001^*^	14.6%
Max_0–4_	Q4	XI.255	2.74	0.069^ms^	8.2%
Max_0–12_	Q2	VII.612	3.57	0.009^*^	3.8%
	Q5	XI.505	3.22	0.027^*^	13.1%
Max_4–12_	Q3	VII.1008	3.48	0.009^*^	3.1%
	Q5	XI.505	8.57	0.000^*^	29.7%
AUC_0–4_	Q4	XI.255	3.74	0.001^*^	9.2%
AUC_0–12_	Q3	VII.1008	3.21	0.015^*^	4.2%
	Q5	XI.505	6.15	0.000^*^	27.3%
AUC_4–12_	Q3	VII.1008	2.58	0.097^ms^	3.3%
	Q5	XI.505	8.27	0.000^*^	31.6%

Time, maximum luminescence time. Max, maximum luminescence value. AUC, area under the luminescence curve. _0–4_, 0−4 h interval. _0–12_, 0−12 h interval. _4–12_, 4−12 h interval. ^*^Statistically significant QTL (*p*-value < 0.05). ^*ms*^Marginally significant QTL (0.05 ≤ *p*-value < 0.15).

**TABLE 2:** Selected candidate genes for each QTL.

**QTL**	**Position (Chr.Kb)**	**Gene**
Q1	II.309	*YPK3, FIG1, FAT1*
Q2	VII.612	*MUP1, LST7, PEF1*
Q3	VII.1008	*GUS1, PIB2, TNA1, APL6*
Q4	XI.255	*GFA1, HSL1*
Q5	XI.505	*SAP190, SET3, KAE1, GAP1, UTH1*

### Candidate Genes Validation Highlight the Importance of tRNAs in TORC1 Activation

Candidate genes were validated by reciprocal hemizygosity analysis (Steinmetz et al., 2002), an approach that consists in the phenotypic comparison of two diploid strains carrying the same genetic background excepting for one allele of the candidate gene. Since the hemizygous strains differ only in one allele (from one or the other parental strain), any phenotypic difference between them is due to that gene. To achieve this, it was necessary to perform the transformations to obtain 34 null mutants (each candidate gene in the two haploid parental strains) and the subsequent crosses to obtain 34 diploid hybrids (17 pairs of hemizygous strains). These strains were evaluated in the same way as the parental strains and the recombinant population, i.e., by measuring the luminescence in microculture conditions by proline to glutamine upshift experiments, and then quantifying the three parameters associated with the luminescence curves.

**FIGURE 2:**
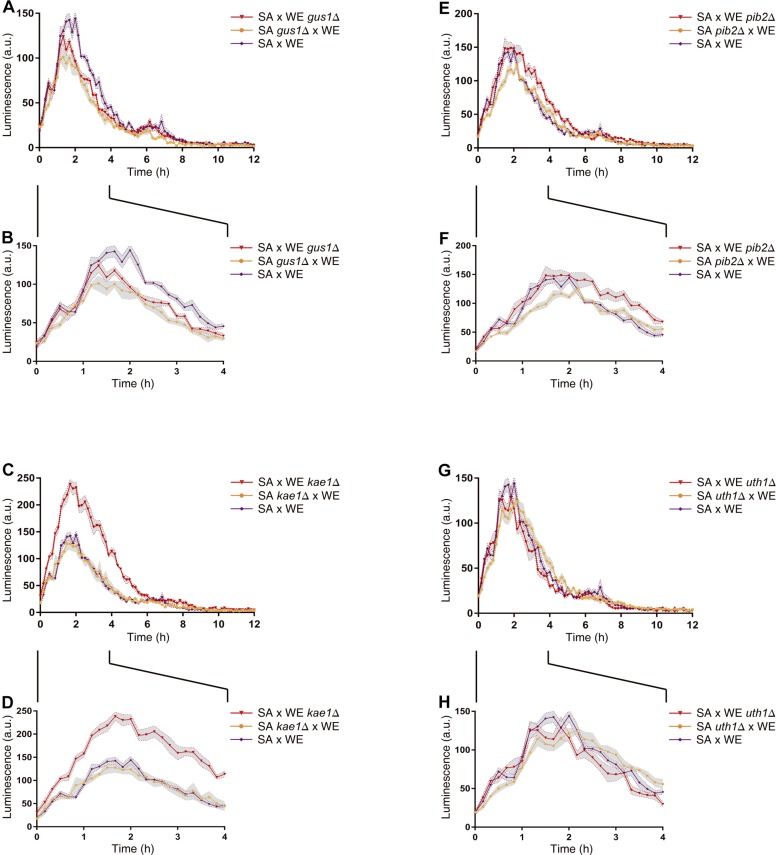
Reciprocal hemizygosity analysis by nitrogen upshift experiments in microculture. Luminescence differences between reciprocal hemizygous strains for the genes *GUS1* **(A,B)**, *KAE1* **(C,D)**, *PIB2* **(E,F),** and *UTH1* **(G,H)** were evaluated in microculture conditions after a pulse of glutamine. The luminescence was recorded until 12 h after the nitrogen pulse **(A,C,E,G)**. Zoom-in of the first 4 h after the nitrogen (proline-to-glutamine) upshift experiment **(B,D,F,H)**. In all panels **(A–H)**, the time 0 h corresponds to the addition of glutamine. Plotted values correspond to the average of three biological replicates, with their standard error represented by shadow regions (mean ± SEM).

In general, the reciprocal hemizygous strains showed a similar luminescence pattern (TORC1 activation) compared to the hybrid strain, with a single peak within the first 4 h of the experiment, and in most cases a slightly lower activation (a minor peak). Considering the reciprocal hemizygous strains that showed statistically significant phenotypic differences, 4 of the 17 genes evaluated were validated: *GUS1* (glutamyl-tRNA synthetase), *KAE1* (ATPse component of the EKC/KEOPS complex and required for t^6^A tRNA modification), *PIB2* (protein of unknown function) and *UTH1* (mitochondrial inner membrane protein) (Figure 2 and Table 3). Then, we analysed for each validated gene the non-synonymous polymorphisms present in the coding sequence to hypothesise the effect of each amino acid change in the protein function (Table 4). Interestingly, two of the four validated genes (*GUS1* and *KAE1*) are involved in tRNAs function.

**TABLE 3:** Parameters associated with the luciferase expression in the reciprocal hemizygous strains.

**Strain**	**Max ± SD (a.u.)**	**Time ± SD (h)**	**AUC_0–12_ ± SD (a.u.)**	**AUC_0–4_ ± SD (a.u.)**
SA × WE	161±7	1.8±0.3	457±16	352±14
SA *gus1*Δ× WE	114±14^*^	1.6±0.2^*ns*^	319±42^*^	254±34^*^
SA × WE *gus1*Δ	137±18^*^	1.4±0.3^*ns*^	379±21^*^	291±16^*^
SA *kae1*Δ× WE	144±25^*^	1.8±0.3^*ns*^	449±97^*^	343±74^*^
SA × WE *kae1*Δ	249±19^*^	1.8±0.1^*ns*^	843±57^*^	647±52^*^
SA *pib2*Δ× WE	133±9^*^	1.9±0.3^*ns*^	421±29^*^	317±22^*^
SA × WE *pib2*Δ	168±23^*^	1.8±0.2^*ns*^	554±40^*^	417±45^*^
SA *uth1*Δ× WE	139±13^*ns*^	1.9±0.5^*ns*^	449±31^*^	334±25^*ns*^
SA × WE *uth1*Δ	145±16^*ns*^	1.6±0.3^*ns*^	398±27^*^	316±27^*ns*^

Max, maximum luminescence value. Time, maximum luminescence time. AUC, area under the luminescence curve. _0–12_, 0−12 h interval. _0–4_, 0−4 h interval. SD, standard deviation. Statistical analysis of these parameters consisted in Welch two sample *t*-tests, which were performed using R software (R Core Team, 2013). ^*^Statistically significant difference between reciprocal hemizygous strains (*p*-value < 0.05). ^*ns*^Non-statistically significant difference between reciprocal hemizygous strains (*p*-value ≥ 0.05). SD, standard deviation.

**TABLE 4:** Analysis of the non-synonymous polymorphic sites in the validated genes.

**Protein**	**Amino acid**		**Position in the protein^a^**	**PROVEAN score**	
	**SA**	**WE**		**SA > WE**	**WE > SA**
Gus1	N176	S176	Part of alpha helix near to the interaction domain with Arc1	1.554	−1.554
	P319	S319	Close to phosphorylation site (T300)	2.655	−2.655^*^
	E321	D321	Close to phosphorylation site (T300)	−2.817^*^	2.817
Kae1	S98	N98	^b^	−0.835	0.835
	L228	I228	Close to substrate binding sites (D194, G209, E213)	−1.834	1.834
Pib2	S67	L67	Close to several phosphorylation sites (S46, S53, T56, S73)	−1.063	1.063
	N446	K446	Close (or belonging) to FYVE domain	2.417	−2.417^+^
	T570	A570	Close to C-terminal extreme (important for TORC1 regulation)	0.209	−0.117
	R588	L588	Close to C-terminal extreme (important for TORC1 regulation)	−1.152	1.243
Uth1	A47	T47	Located in an alanine-rich region	0.694	−0.829
	T54	N54	Located in an alanine-rich region	−0.646	0.911
	S101-S103	−	Located in a poly-serine region	−4.364^*^	4.835
	F127	S124	^b^	3.933	−3.983^*^

^*a*^Position in the protein according to the laboratory strain (S288c). ^*b*^No special features identified. ^*^Variant predicted as deleterious (PROVEAN score < 2.500). ^+^Variant not predicted as deleterious but very close to the statistical cut (PROVEAN score < 2.400).

**FIGURE 3:**
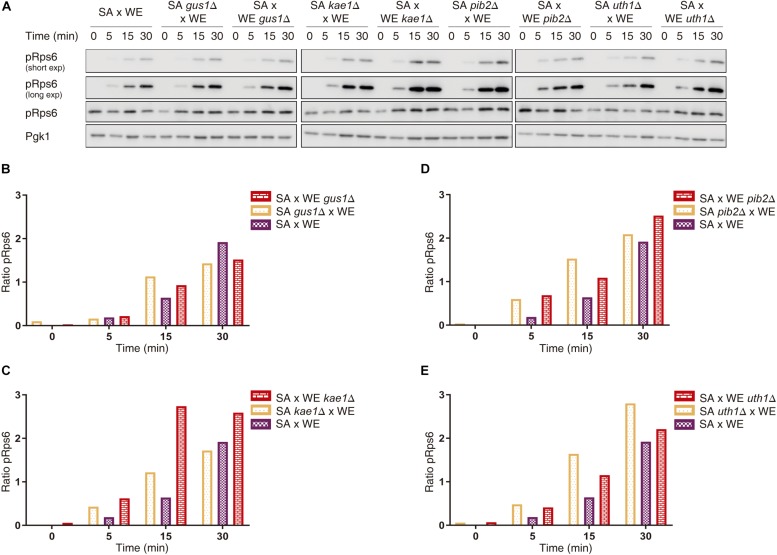
Quantification of Rps6 phosphorylation for reciprocal hemizygous strains. **(A)** Western blot for the reciprocal hemizygous strains. Phosphorylated (pRps6) and total Rps6 protein levels were evaluated using specific antibodies. Time 0 min corresponds to the addition of glutamine. Pgk1 was used as a loading control. **(B–E)** Quantification of Rps6 phosphorylation for the genes *GUS1* **(B)**, *KAE1* **(C)**, *PIB2* **(D),** and *UTH1* **(E)**. The “Ratio pRps6” is the ratio between pRps6 band intensity and the total Rps6 band intensity, normalised by the mean value of all the ratios obtained.

We further confirmed the effect of the validated genes over TORC1 activity by assessing Rps6 phosphorylation by Western blot, a method that directly evaluates TORC1 activation given that Rps6 is phosphorylated in response to the activation of TORC1 (Gonzalez et al., 2015). In the case of the reciprocal hemizygotes for the *GUS1* gene, there was no phenotypic difference 30 min after the glutamine pulse (Figure 3 and Supplementary Data Sheet 1). However, for *KAE1*, *PIB2*, and *UTH1* genes we confirmed the results obtained by luminescence curves, in which the reciprocal hemizygous strains carrying the SA alleles for *KAE1* and *PIB2* genes showed the greater TORC1 activation, whereas hemizygous strain containing the WE allele of *UTH1* gene showed a greater activation.

### The Allelic Variants Identified Affects the Fermentation Kinetics

Due to the relationship that has been reported between TORC1 pathway activation and wine fermentation in nitrogen deficient conditions (Tesniere et al., 2015), we decided to evaluate the growth parameters of the reciprocal hemizygous strains under microculture conditions. We extracted from the growth curves the lag time, growth rate and efficiency of proliferation (population density change). We assayed the hemizygous strains for the validated genes (*GUS1*, *KAE1*, *PIB2*, and *UTH1*) in three synthetic musts containing different nitrogen concentrations (60, 140, and 300 mgN/L), which correspond to limited, standard and excessive nitrogen conditions, respectively. The results showed phenotypic differences between the hemizygous strains (Supplementary Table 3), observing that strains carrying the WE allele of *GUS1*, *KAE1*, and *PIB2* genes showed higher growth rates, with the opposite effect for the *UTH1* gene.

**FIGURE 4:**
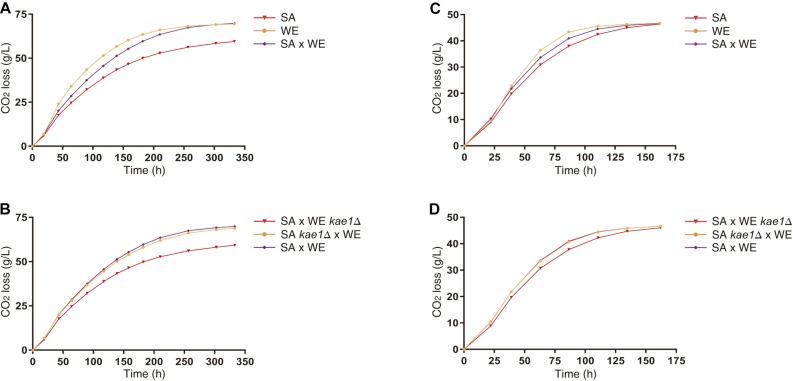
Fermentation of parental and reciprocal hemizygous strains for *KAE1* gene. The differences in CO_2_ loss between parental strains **(A,C)** and reciprocal hemizygous strains **(B,D)** for the *KAE1* gene were evaluated in microscale fermentations in SM60 **(A,B)** and SM300 **(C,D)**. Plotted values correspond to the average of three biological replicates, with their standard error represented by bars (mean ± SEM).

To study in detail the effect of these variants on the fermentative capacities of *S. cerevisiae*, microscale fermentations (10 mL) were carried out under limiting (60 mgN/L) and non-limiting (300 mgN/L) nitrogen conditions. The reciprocal hemizygous strains for the *KAE1* gene showed phenotypic differences in their fermentative profile in both conditions (Figure 4), with the reciprocal hemizygote carrying the WE allele having a greater CO_2_ loss (an indicator of the fermentation activity), which coincides with the observed phenotypes in the parental strains (the WE strain showed a higher CO_2_ loss in comparison with the SA strain). The reciprocal hemizygous strains for *GUS1*, *PIB2* and *UTH1* did not show phenotypic differences in their fermentation kinetics (Supplementary Figure 3). Altogether, our results demonstrated that the differential activation of the TORC1 pathway caused by different *KAE1* alleles also impacts the fermentative capacities of yeast.

## Discussion

Although the distal and proximal effectors downstream TORC1 are well known, it is not fully understood the mechanism by which TORC1 is activated by nitrogen sources (Conrad et al., 2014; Gonzalez and Hall, 2017). In the present study, we performed a linkage analysis utilising phenotypic data from a recombinant yeast population, which was phenotyped for TORC1 activation using nitrogen-upshift experiments, transferring the cells from proline to glutamine. This population was derived from a cross between two strains (SA and WE) that previously showed great phenotypic differences for TORC1 activation (Kessi-Perez et al., 2019), and has been successfully used in several studies to determine the genetic basis of different phenotypes related to the wine fermentation process, such as nitrogen sources consumption (Jara et al., 2014), glycerol and acetic acid production (Salinas et al., 2012), and resistance to fungicides and fermentation kinetics (Kessi-Perez et al., 2016).

This approach allowed us to map several QTLs, being a QTL in chromosome XI (XI.505) the most significant peak mapped (Figure 1 and Table 1). From these QTLs, 17 candidate genes were selected (Table 2), and to verify the participation of each candidate gene in the studied phenotype, we performed a reciprocal hemizygosity analysis (Steinmetz et al., 2002). This validation approach has the advantage that the phenotypic differences in the hemizygous strains are not the result of interactions between the genetic background of the hybrid and the deleted allele dominance (haploinsufficiency), since both hemizygotes have the same genetic background, except for the locus under study (Kim and Cunningham, 2015).

The results obtained for the hemizygous strains (Figure 2 and Table 3) allowed the validation of *GUS1*, *KAE1*, *PIB2*, and *UTH1* genes as responsible for the phenotypic differences observed between SA and WE strains. Although the phenotypic differences caused by the alleles of these genes were small compare to the phenotypic differences between parental strains (Supplementary Figure 1), this result is consistent with phenotypes of complex inheritance (complex traits), which are the result of many loci with combined small effects (Mackay et al., 2009). However, we mapped a major effect QTL in chromosome XI (explaining over 30% of the phenotypic variance) (Table 1), which is not usual in linkage approaches, reinforcing the importance of our phenotyping strategy. Our phenotyping strategy also poses novel questions, such as what is the biological meaning of the second luminescence peak observed between 6 and 12 h for the WE strain (Supplementary Figure 1). This phenomenon was present in the haploid WE strain and some of the haploid segregant derived from the SA×WE hybrid, suggesting possible genetic determinants underlying this observation, which was also previously described by Kessi-Perez et al. (2019). Further analyses are required to study this second peak and identify its genetic determinants.

These results were also confirmed through Rps6 phosphorylation, which allows a direct TORC1 activation evaluation (Gonzalez et al., 2015) (Figure 3). Except for *GUS1*, we observed a correlation between the indirect method (luciferase) and direct detection (Western blot) of TORC1 activation. This, considering that both methods are not comparable since the first one is a transcriptional reporter and the second one is measuring a post-translational modification, respectively (Kessi-Perez et al., 2019). Considering the reported evidence and the results obtained in this work, it is possible to hypothesise a possible role for the validated genes in TORC1 activation, as we summarised in Supplementary Figure 4, where these genes could be interacting directly or indirectly with the TORC1 complex.

Among the validated genes, reciprocal hemizygous strains for *KAE1* presented the greatest phenotypic differences for TORC1 activation (Figure 2). This gene encodes a highly conserved ATPase of the HSP70/DnaK family that is part of EKC/KEOPS complex, which in turn is involved in the t^6^A modification of tRNAs (Daugeron et al., 2011; Srinivasan et al., 2011). Although neither *KAE1* nor its human counterpart (*OSGEP*) have been directly associated with TORC1 or mTORC1 regulation, there is evidence of the relationship between the tRNAs and the activation of TORC1, which in all cases appears to be EGOC-independent (Huynh et al., 2010; Rojas-Benitez et al., 2015; Thiaville and de Crecy-Lagard, 2015; Kamada, 2017; Otsubo et al., 2018).

In the case of *S. cerevisiae*, the aminoacyl-tRNA synthetases regulate TORC1 pathway activity by a proposed mechanism in which free tRNAs inhibit TORC1 activity (Kamada, 2017). In addition, it has been reported that mutants for t^6^A mcm^5^s^2^U modifications are hypersensitive to the TORC1 inhibition by rapamycin. Thus, they could be key for the pathway activity, especially t^6^A (Scheidt et al., 2014; Thiaville and de Crecy-Lagard, 2015; Thiaville et al., 2016). Similarly, in *Schizosaccharomyces pombe* it has been observed that proteins involved in the expression or modification of tRNAs (e.g., aminoacyl-tRNA synthetases) are necessary for TORC1 activity, and in particular that overexpression of tRNA precursors (pre-tRNAs) prevents the inhibition of TORC1 after depletion of nitrogen source (Otsubo et al., 2018). In metazoans, some studies point out in the same direction. In *Drosophila*, Prpk/Tcs5 protein (Bud32 in *S. cerevisiae*), another member of EKC/KEOPS complex, is involved in dTORC1 activation (Ibar et al., 2013). Furthermore, a change in the proportion of t^6^A-modified tRNAs or in the levels of Kae1/Tcs3 (Kae1 in *S. cerevisiae*) regulates dTORC1 activity (Rojas-Benitez et al., 2015). Finally, in human fibroblasts it has been observed that a mutation causing tRNA accumulation in the nucleus leads to a reduction in mTORC1 activity, suggesting that charged tRNA activates the pathway (Huynh et al., 2010).

The idea that tRNAs are involved in the regulation of TORC1 activity is strengthened by the validation of *GUS1* gene in this work, which encodes for the glutamyl-tRNA synthetase (GluRS) and forms a complex with Arc1 and Mes1 (methionyl-tRNA synthetase) (Deinert et al., 2001; Galani et al., 2001). Overexpression of *GUS1* in a laboratory background (S288c strain) causes increased resistance to rapamycin (Butcher et al., 2006). Although *GUS1* has not been previously related to TORC1 regulation in *S. cerevisiae*, there are antecedents of the relationship between its human counterpart (*EPRS*) and mTORC1 (Arif and Fox, 2017; Arif et al., 2017). In this sense, recent reports demonstrate that TORC1 (and mTORC1) effectors can also function as upstream regulators, working as homeostatic feedback loops, which means that new TORC1 regulators may be found among newly and previously identified effectors (Eltschinger and Loewith, 2016).

Meanwhile, although Pib2 has been considered a protein of unknown function which was only known to contain a FYVE domain and similarity to Fab1 and Vps27 (Burd and Emr, 1998), the results agree with the relationship between Pib2 and TORC1 activation that several authors have raised in recent years (Kim and Cunningham, 2015; Michel et al., 2017; Tanigawa and Maeda, 2017; Varlakhanova et al., 2017; Ukai et al., 2018; Sullivan et al., 2019). In addition, other evidences point out the possible involvement of Pib2 in TORC1 activation are its possible location in the vacuolar membrane and the phenotype of the null mutant in the laboratory strain (S288c), which showed a decreased resistance to rapamycin (Xie et al., 2005). It has been postulated that Pib2 could be a glutamine sensor that activate TORC1 in an EGO-independent way (Kim and Cunningham, 2015; Ukai et al., 2018), which fully agrees with our results and validates the approach used in this study.

Finally, the null mutant of *UTH1* in the laboratory W303 strain has an increased resistance to rapamycin (Kissova et al., 2004; Ritch et al., 2010). This gene encodes a protein member of the SUN family, located in the inner mitochondrial membrane and involved in the response to oxidative stress (Bandara et al., 1998) and cell wall biogenesis (Ritch et al., 2010). The ability of Uth1 to regulate TORC1 has been previously described (Lin et al., 2016), and it may be related to the cross-regulation previously reported for the Rho1 kinase (a central member of the cell wall integrity pathway) (Rodkaer and Faergeman, 2014). Interestingly, *UTH1* was the only gene in which the reciprocal hemizygote with the WE allele showed the highest TORC1 activation, which could be related to the increased resistance to rapamycin of the *UTH1* null mutant.

We also analysed the polymorphisms affecting the proteins encoded by the validated genes (Table 4). In the case of GluRS (*GUS1*), the crystallographic structure is available, specifically the interaction site with Arc1p (Simader et al., 2006a, b). The polymorphism present at position 176 (N176 in the SA strain and S176 in the WE strain) is part of an alpha helix very close to that interaction site. However, PROVEAN analysis point out other two polymorphisms as the most likely cause of the observed phenotypic differences (Table 4). For Pib2, the possible effect of the four polymorphisms found can be hypothesised. The polymorphism present at position 67 (S67 in the SA strain and L67 in the WE strain) was not predicted as deleterious by the PROVEAN analysis, however, this polymorphism is in a region rich in serine residues that can be phosphorylated (Holt et al., 2009). Thus, it can be speculated that a serine residue at this position could be phosphorylated under certain conditions in the SA strain. Similarly, the polymorphism present at position 446 (N446 in the SA strain and K446 in the WE strain) could influence the functionality of the FYVE domain present in the protein, variant that was almost predicted as deleterious by the PROVEAN analysis (Table 4). Finally, the other two polymorphisms (positions 570 and 588) are located near the C-terminal end of the Pib2 protein, which has been described as important for the regulation of TORC1 activity (Kim and Cunningham, 2015; Michel et al., 2017).

Finally, utilising synthetic musts with different nitrogen concentrations, we performed growth curves for the reciprocal hemizygous strains of the validated genes (Supplementary Table 3). The strains carrying the WE allele of the genes *GUS1*, *KAE1*, and *PIB2* tend to have a higher growth rates, being the opposite case for the *UTH1* gene. This correlates with the observations made in nitrogen upshift experiments (Figure 2 and Table 3), in which also the *UTH1* gene showed an inverse behaviour respect to the other genes. Meanwhile, only *KAE1* reciprocal hemizygous strains showed phenotypic differences in microscale fermentations (Figure 4 and Supplementary Figure 3), probably due to the strong effect of this gene over TORC1 activation (Figure 2). In addition, *KAE1* gene is highly pleiotropic, impacting multiples phenotypes including the alcoholic fermentation (Figure 4). It is interesting to note that, although the phenotypic differences between hemizygous strains exist under non-limiting conditions, they seem to increase in the nitrogen-limited environment (Figure 4). This reinforce the role of the TORC1 pathway during the alcoholic fermentation, specially under low nitrogen conditions (Tesniere et al., 2015). Interestingly, the hemizygous strain carrying the *KAE1*^*SA*^ allele showed a reduced fermentation rate compared to the strain with WE allele (Figure 4). This result may seem counterintuitive, since *KAE1*^*SA*^ allele showed a higher TORC1 activation, however, this kind of antagonistic effect have been also observed for a loss-of-function variant of *RIM15*, a gene involved in sporulation and stress response (also under TORC1 control), and that also impacts the fermentative performance (Kessi-Perez et al., 2016). We have hypothesised that *KAE1*^*SA*^ allele causes a hypersensitive response to nitrogen presence, rapidly depleting the preferred nitrogen sources present in the fermentation medium and reducing the fermentation progression. Similarly, *KAE1*^*WE*^ allele causes an impaired detection of nitrogen presence/absence by the cell, causing less activation of TORC1 when nitrogen sources are added to the medium, but also a less deactivation of the pathway when the nitrogen sources are depleted. This effect can cause less stress response (like the loss-of-function variant of *RIM15*), which in turn can lead to yeast cells to continue fermenting instead of activating the stress response program. Further studies are required to test these hypotheses at the molecular level, such as deactivation experiments of TORC1 by a nitrogen downshifts or a rapamycin treatment.

## Conclusion

In conclusion, we identified several QTLs explaining the natural variation in TORC1 activation. From these QTLs we validated *GUS1*, *KAE1*, *PIB2*, and *UTH1* genes as responsible for this phenotype in *S. cerevisiae*. In addition, our results highlight the importance of tRNA processing in TORC1 activation, specifically t^6^A modification and aminoacylation, which ultimately impact on the fermentative capacities of the assayed strains, especially in low-nitrogen conditions.

## Data Availability

The datasets generated for this study are available on request to the corresponding author.

## Author Contributions

EK-P, FS, JG, MH, LL, and CM designed and funded the research. EK-P, YS, and AG performed the lab experiments and analysed the experimental data. EK-P and FS wrote the manuscript with insight from all the authors.

## Conflict of Interest Statement

The authors declare that the research was conducted in the absence of any commercial or financial relationships that could be construed as a potential conflict of interest.

## Footnotes

1 http://www.moseslab.csb.utoronto.ca/sgrp/blast_new/

2 http://web.expasy.org/translate/

3 https://www.ebi.ac.uk/Tools/msa/clustalo/

4 http://provean.jcvi.org/index.php

5 www.yeastgenome.org
