# Supplementary figures and images for: KAE1 Allelic Variants Affect TORC1 Activation and Fermentation Kinetics in Saccharomyces cerevisiae
Source: Front Microbiol. 2019 Jul 31;10:1686. doi: 10.3389/fmicb.2019.01686 (PMC6685402; doi:10.3389/fmicb.2019.01686)

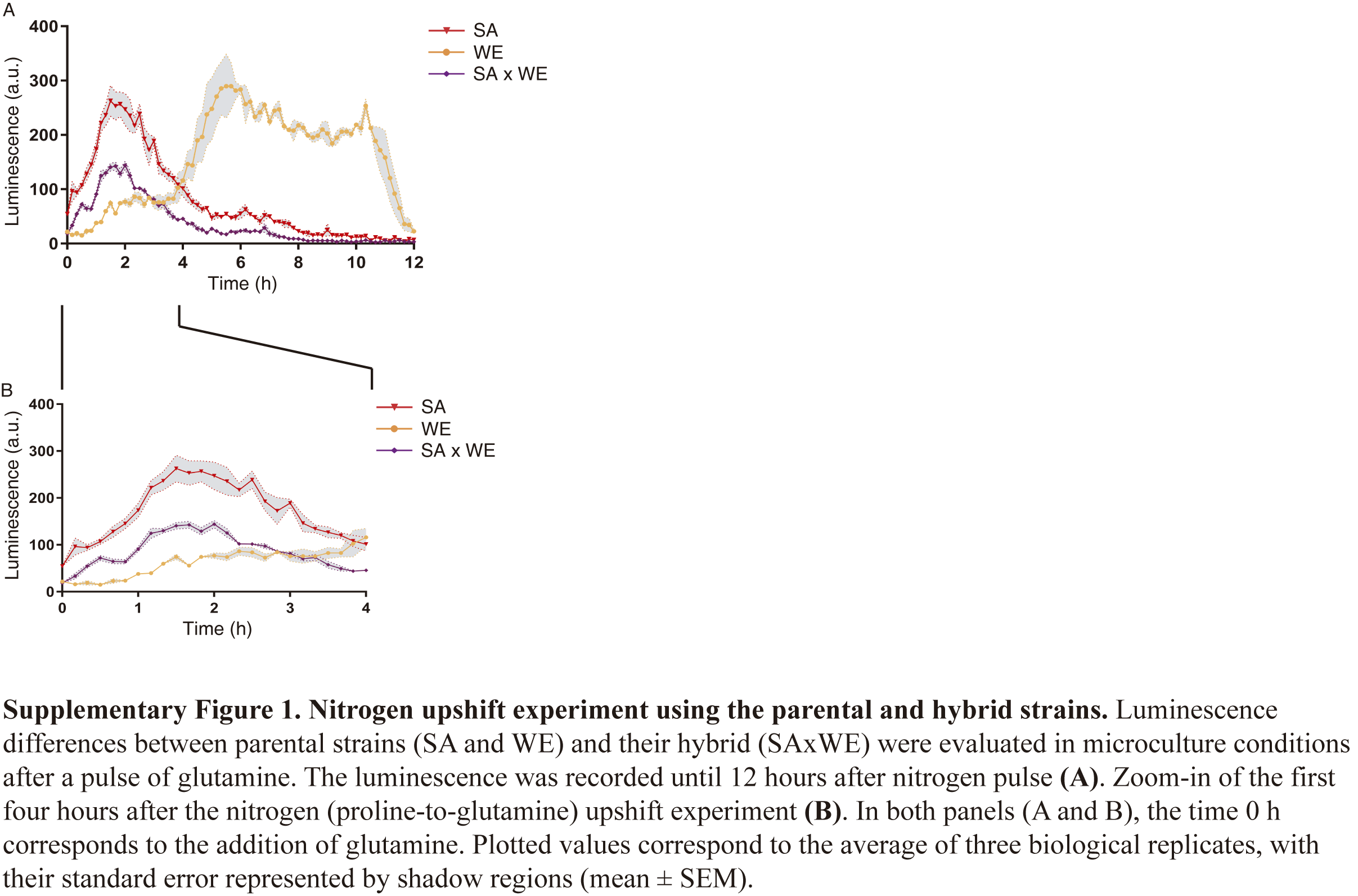

Supplement: Supplementary file 1 [file Image_1.TIF]

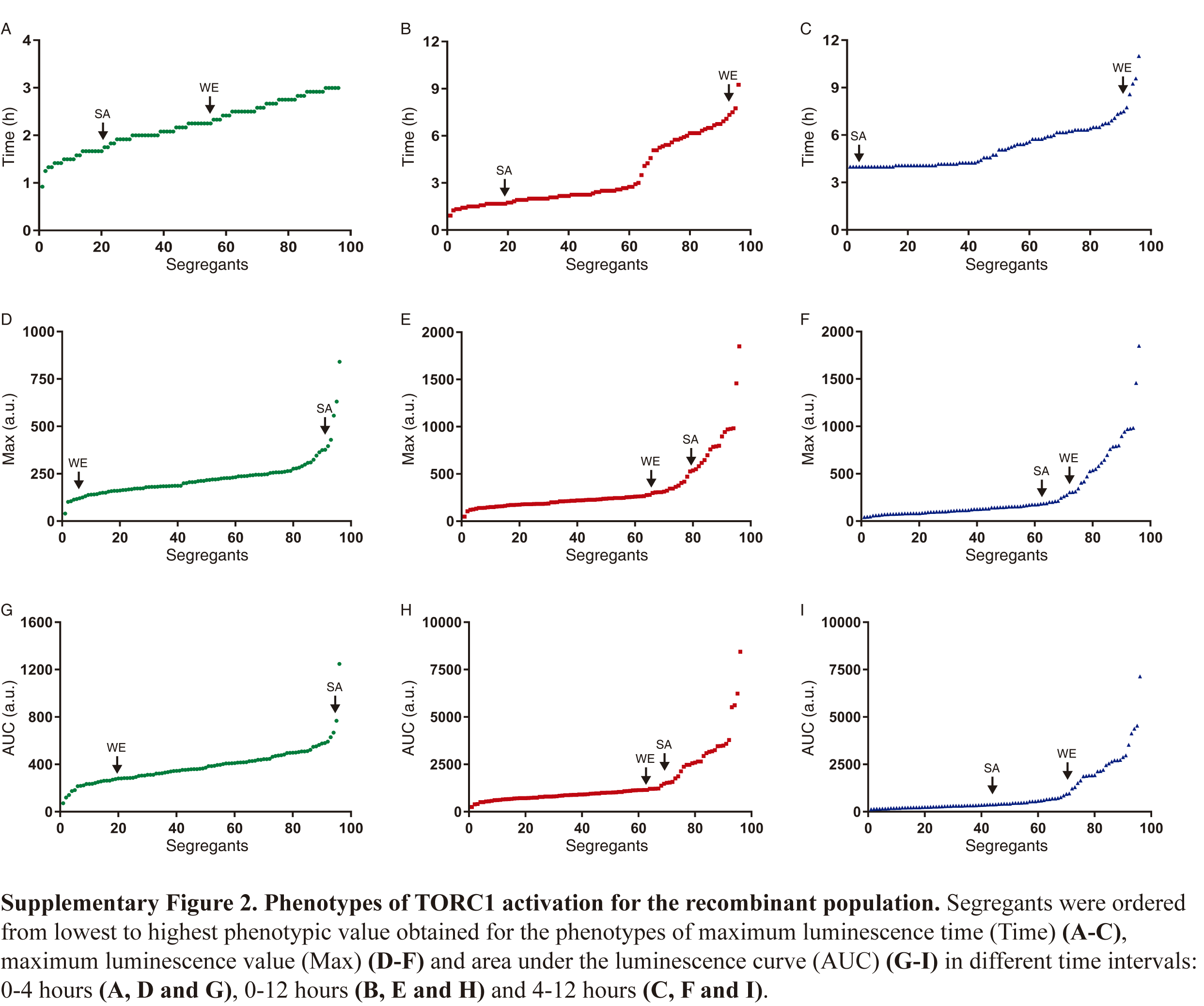

Supplement: Supplementary file 2 [file Image_2.TIF]

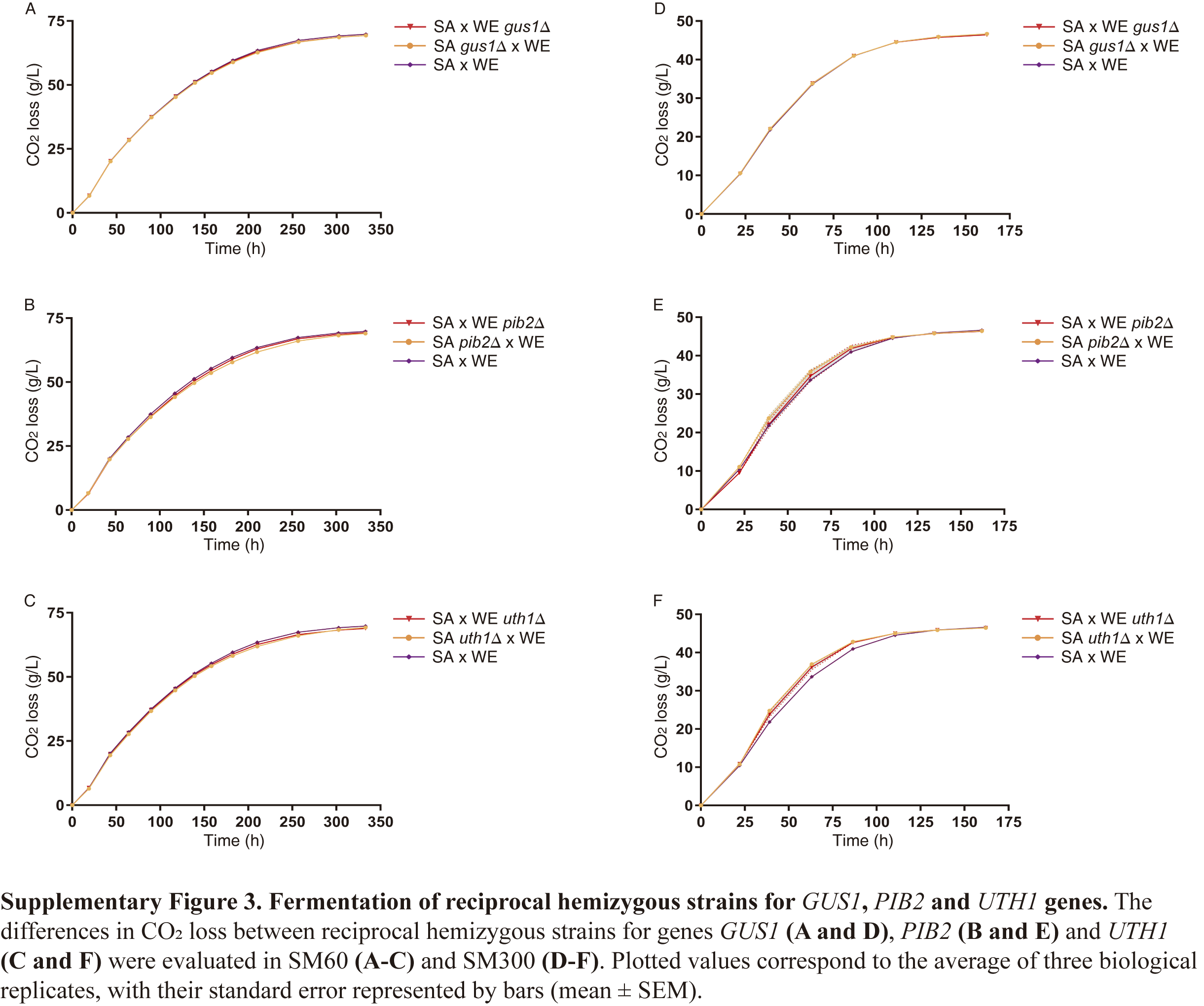

Supplement: Supplementary file 3 [file Image_3.TIF]

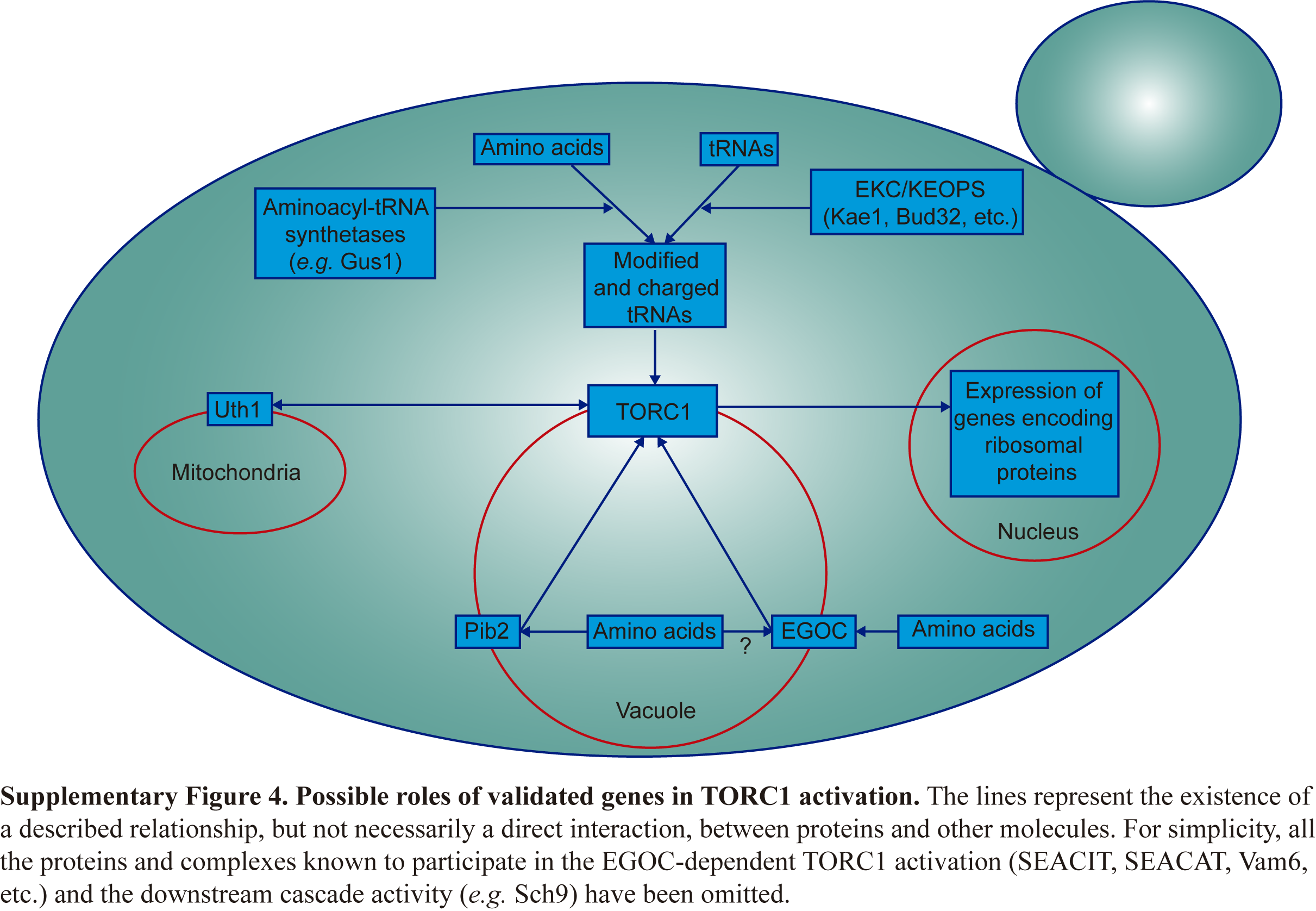

Supplement: Supplementary file 4 [file Image_4.TIF]
