# Supplementary material for: KAE1 Allelic Variants Affect TORC1 Activation and Fermentation Kinetics in Saccharomyces cerevisiae
Source: Front Microbiol. 2019 Jul 31;10:1686. doi: 10.3389/fmicb.2019.01686 (PMC6685402; doi:10.3389/fmicb.2019.01686)
Supplement: Supplementary file 5 [file Data_Sheet_1.ZIP › File_information.docx]

**Supplementary File 1. Full scan of the entire original gels from Western blot.** The images were generated not from films but from a chemiluminescence machine used to reveal. Each image corresponds to a different exposition time, numbered from one to seven. Starting from the top, the first three gels are for pRps6, the next three are for Rps6 and the last three are for Pgk1.
